# Capillary refill time is a poor predictor of 30-day mortality: an observational cohort study

**DOI:** 10.1186/1757-7241-21-S2-A20

**Published:** 2013-09-09

**Authors:** Monija Mrgan, Dorte Rytter, Mikkel Brabrand

**Affiliations:** 1 Department of Cardiology, Sydvestjysk Sygehus, Finsensgade 35, 6700 Esbjerg, Denmark; 2 Department of Medicine, Sygehus Lillebælt, Dronningensgade 97, 7000 Fredericia, Denmark

## Background

Capillary refill time (CRT) was introduced in 1947. In the 1980`s, it was proposed as one of five elements in the Trauma Score and defined as two seconds or less in all adult patients. An alternative definition (sex and age dependent) has been introduced by Schriger and Baraff.

We performed a prospective observational cohort study to assess the relationship between CRT and 30-day mortality.

## Methods

The study originates from the medical admission unit at Sydvestjysk Sygehus, Esbjerg from 2 October 2008 to 19 February 2009. All acutely admitted adult patients (age 15 and older)were included and the nurse recorded and reported the vital signs (including CRT). The primary outcome was 30-day all-cause mortality. To ensure complete follow-up, data on the endpoint was extracted from the Danish Person Register. Difference between continuous data was analyzed using Wilcoxon Rank Sum Test and categorical data were compared using chi-squared test. We performed multivariable logistic regressions to identify CRT as an independent predictors of 30-day mortality controlling for other vital signs, sex and age.

## Results

A total of 3,046 patients were enrolled and CRT was measured on 1,935 (63.5 %). Patients with a CRT ≤ 1 had a 30-day mortality of 3.8 % compared to patients with a CRT ≥ 5 (18.2 %). Patients with an abnormal CRT according to the Trauma Score had a 30-day mortality of 8.6 % versus 5 % (p = 0.002). Abnormal CRT according to the Schriger and Baraff's definition resulted in a 30-day mortality of 5.5 % versus 6.3 % (p = 0.51). Logistic regression showed CRT not to be an independent predictor of 30-day mortality, neither as a continuous variable, nor by either definition.

## Conclusion

CRT is associated with mortality, however, we were only able to show this in univariable analyses and only for the Trauma Score definition. When performing multivariable logistic regression controlling for the other vital signs, we were unable to show any association.

Our data show that CRT is a poor vital sign and we discourage use in the clinical setting.

